# Imaging of the oesophagus: beyond cancer

**DOI:** 10.1007/s13244-017-0548-3

**Published:** 2017-03-17

**Authors:** Thomas Marini, Amit Desai, Katherine Kaproth-Joslin, John Wandtke, Susan K. Hobbs

**Affiliations:** Department of Imaging Sciences, University of Rochester Medical Center, 601 Elmwood Ave, Box 648, Rochester, NY 14642 USA

**Keywords:** Oesophagus, Non-malignant, Stricture, Dilatation, Rupture

## Abstract

**Abstract:**

Non-malignant oesophageal diseases are critical to recognize, but can be easily overlooked or misdiagnosed radiologically. In this paper, we cover the salient clinical features and imaging findings of non-malignant pathology of the oesophagus. We organize the many non-malignant diseases of the oesophagus into two major categories: luminal disorders and wall disorders. Luminal disorders include dilatation/narrowing (e.g. achalasia, scleroderma, and stricture) and foreign body impaction. Wall disorders include wall thickening (e.g. oesophagitis, benign neoplasms, oesophageal varices, and intramural hematoma), wall thinning/outpouching (e.g. epiphrenic diverticulum, Zenker diverticulum, and Killian-Jamieson diverticulum), wall rupture (e.g. iatrogenic perforation, Boerhaave Syndrome, and Mallory-Weiss Syndrome), and fistula formation (e.g. pericardioesophageal fistula, tracheoesophageal fistula, and aortoesophageal fistula). It is the role of the radiologist to recognize the classic imaging patterns of these non-malignant oesophageal diseases to facilitate the delivery of appropriate and prompt medical treatment.

***Teaching Points*:**

• *Nonmalignant oesophageal disease can be categorised by the imaging appearance of wall and lumen*.

• *Scleroderma and achalasia both cause lumen dilatation via different pathophysiologic pathways*.

• *Oesophageal wall thickening can be inflammatory, neoplastic, traumatic, or vascular in aetiology*.

## Introduction

**Fig. 1:**
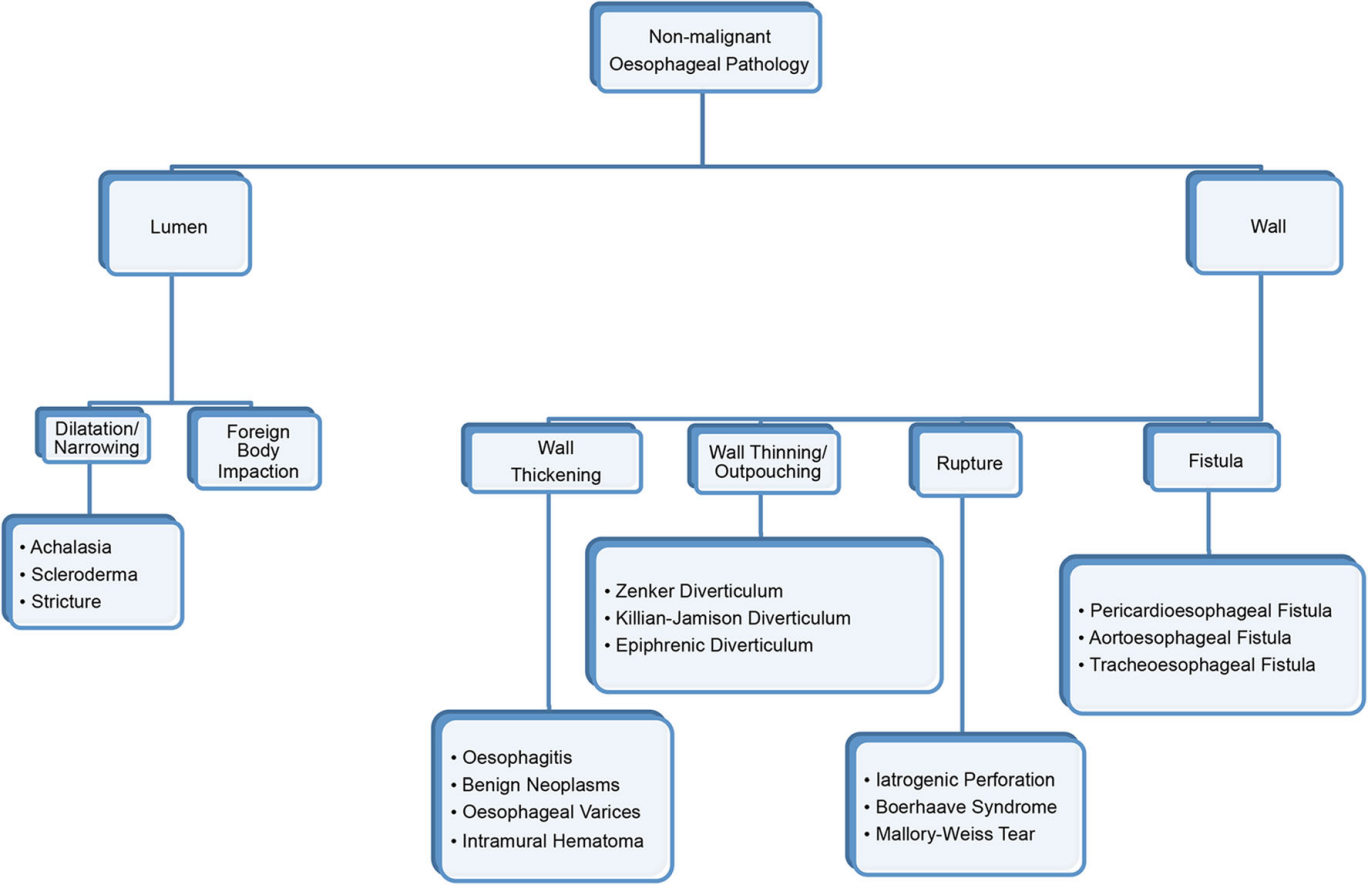
Our conceptual organization of oesophageal pathology

Although they are often eclipsed by oesophageal cancer, it is important to think about the many non-malignant oesophageal conditions which may be seen on imaging. We have organised the non-malignant diseases of the oesophagus into two major categories for pedagogical purposes: disorders involving lumen and disorders involving the wall. Luminal disorders can be then divided into categories of dilatation/narrowing and foreign body impaction. Wall disorders encompass a broader range of pathology and are divided into categories of wall thickening, wall thinning, wall rupture, and fistula formation. This classification scheme for nonmalignant disorders of the oesophagus and the common disorders in each category can be seen in Fig. 1. It is important to note that luminal and wall disorders may overlap in many cases; therefore, this scheme is based on the most obvious presenting imaging features.

## Overview of oesophageal imaging

Multiple imaging modalities are used in the evaluation of oesophageal pathology, including computed tomography (CT), barium oesophagography, endoscopic ultrasound (EUS), and 18-Fluoro-deoxyglucose positron emission tomography (FDG-PET) [1, 2]. Barium esophagography is a useful initial imaging modality for the diagnosis of patients with dysphagia, reflux, motility disorders, or perforation. For an intact oesophagus, thick and thin barium are used as contrast; however, if perforation is suspected, a diluted water-soluble contrast agent is typically used followed by dilute barium if no perforation is seen [3].

If pathology is detected during esophagography, CT imaging is often used to clarify findings and define anatomy; it can be particularly helpful in cases of suspected masses as barium esophagography cannot readily define disease extent outside the mucosa. CT imaging can also show wall thickness and evaluate for mediastinal involvement/general extent of disease beyond the mucosa. Importantly, CT imaging can be rapidly performed and is typically well tolerated by patients; this is especially important in patients who are unable to fully participate in a barium swallow study [4]. If oesophageal pathology is suspected, CT should be performed with both intravenous contrast (to assess for enhancing lesions) as well as oral contrast given just prior to imaging (to assist in the evaluation of the oesophageal lumen). If the patient is at high risk for aspiration, water can be used instead of oral contrast to dilate the oesophageal lumen.

If oesophageal pathology is confirmed or highly suspected, endoscopic ultrasound is often utilized to evaluate a specific region of interest as it offers detailed visualization of the layers of the oesophageal wall. In addition, this imaging modality can perform direct biopsy of suspicious lesions, including adjacent lymphadenopathy if present [5]. Limitations of this imaging modality include its operator dependence, the semi-invasive nature of the procedure, and the need to use moderate sedation.

Finally, FDG-PET imaging is typically reserved for cases that are known to be malignant and where evaluation is needed for distant metastasis or to delineate between malignant and nonmalignant aetiologies. It will not be discussed in this paper.

## Anatomy and physiology of the oesophagus

**Fig. 2:**
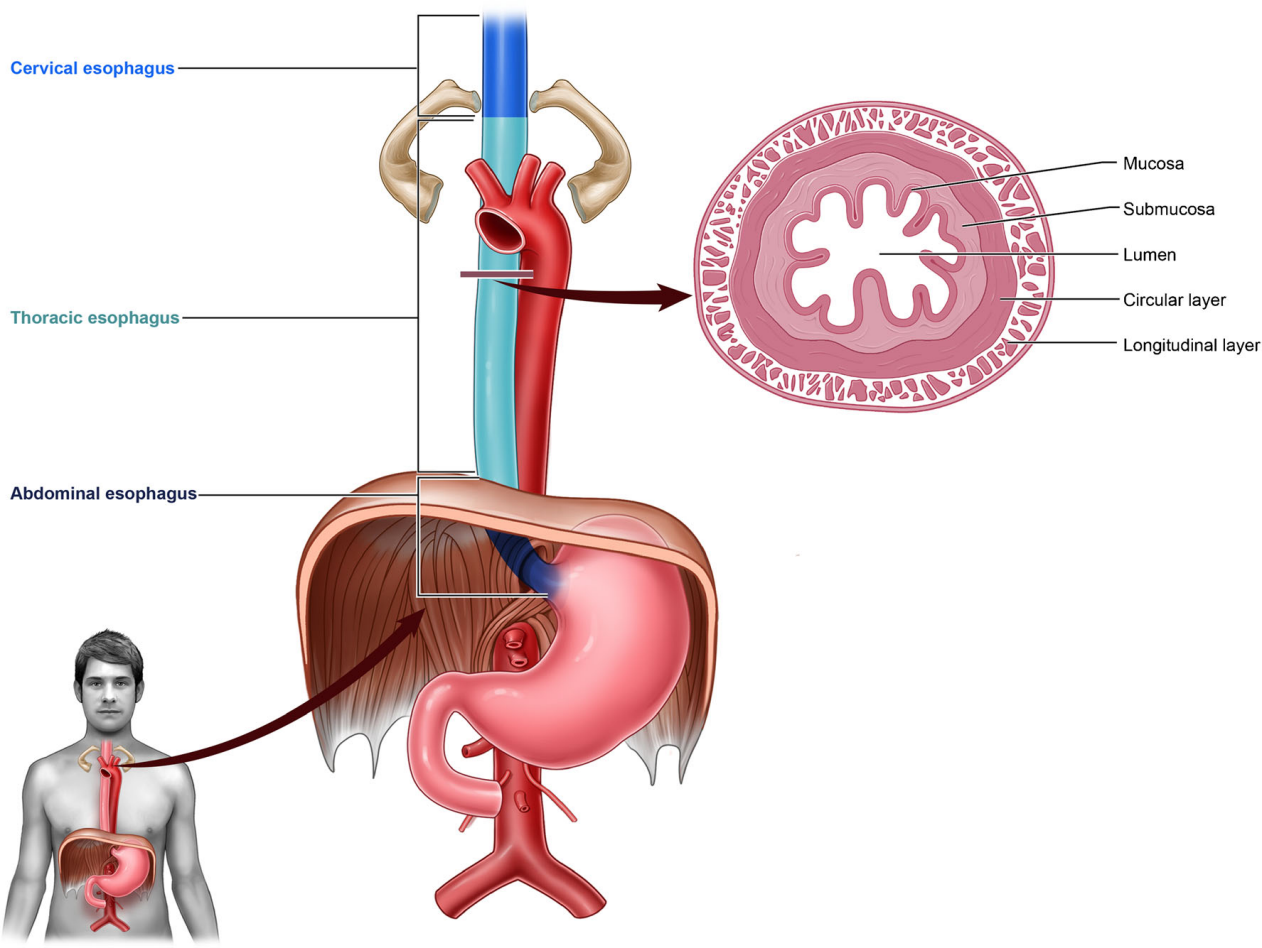
Anatomy and histology of the oesophagus

The oesophagus is a tubular structure that extends from the pharynx to the stomach. It can be divided into 3 anatomic regions: cervical, thoracic, and abdominal [6, 7]. Details of oesophageal anatomy are included in Fig. 2 and Table 1. Of note, superior to the carina, the anterior thoracic oesophagus abuts the posterior trachea and is flanked by the aorta on the left side and the azygos vein on the right. Inferior to the carina, the thoracic oesophagus is just posterior to the left mainstem bronchus and the left atrium. Normal oesophageal peristalsis propels food from the cervical to the abdominal oesophagus via primary and secondary contractions. Primary contractions are the initial waves that propel food through the oesophagus while secondary contractions help move any additional food boluses unmoved by primary contractions [8]. In addition, it is important recognize the natural physiologic points of oesophageal narrowing which occur at the levels of the cricoid cartilage and at the diaphragm, as these are common places of foreign body impaction.

**Table 1:** Salient features of oesophageal anatomy

	Origin	End	Arterial Supply	Venous Drainage
Cervical	Pharyngoesophageal junction (C6)	Suprasternal notch	Inferior thyroid artery	Azygos veins
Thoracic	Suprasternal notch	Diaphragmatic hiatus (T10)	Oesophageal arteries	Left gastric and Portal veins
Abdominal	Diaphragmatic hiatus (T10)	Stomach (T11)	Left gastric and Phrenic arteries	Azygos veins

## Luminal disorders

**Fig. 3:**
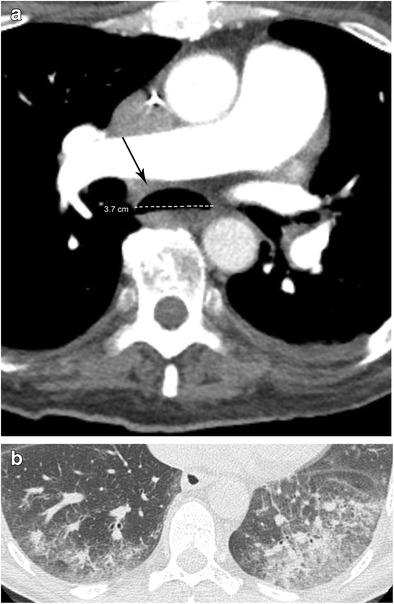
Scleroderma. **a** CT angiogram of the chest in soft tissue window demonstrates a dilated oesophageal lumen. **b** Chest CT of the same patient in lung window demonstrates bilateral dependent areas of ground glass opacity and reticulation with relative subpleural sparing observed in the setting of chronic aspiration

Pathology of the oesophageal lumen is broad in nature and can involve disorders of smooth muscle innervation, scarring, and foreign body impaction. Evidence suggests (with one exception) that any segment of the oesophagus containing air with a diameter of >10 mm should be considered abnormal (Fig. 3a). The exception to this rule is the portion of the oesophagus between the cardiac ventricles and the lower oesophageal sphincter; in this segment, dilatation >15 mm should be considered abnormal [9]. Observation of an air-fluid level is abnormal, but food material within the oesophagus is not considered abnormal. On chest x-ray, we evaluate distention subjectively as there are no established guidelines. However, as a general rule, it is considered abnormal if the oesophageal lumen approaches the luminal size of the trachea for about one third of the oesophageal length. A soft tissue density that distorts the normal mediastinal contour in the region of the oesophagus is also abnormal and further evaluation with either another imaging modality or direct visualization is warranted.

### Luminal disorders due to dilatation and narrowing

#### Achalasia

**Fig. 4:**
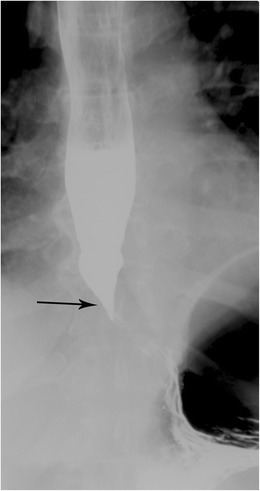
Achalasia. Esophagram in anterior-posterior (AP) projection demonstrates a dilated thoracic oesophagus with smooth tapering distally - the classic “bird beak” appearance of achalasia

Achalasia is caused by the destruction of ganglion cells within the lower oesophageal sphincter (LES) and myenteric plexus stemming from inflammation. Loss of the ganglion cells leads to an overall decreased inhibitory neuronal signal to the smooth muscle resulting in impaired primary and secondary peristalsis and increased tone of the LES. The combination of impaired oesophageal motility and distal narrowing secondary to increased LES tone ultimately results in dilatation of the lumen [10]. The classic manifestation of achalasia on imaging is the “bird beak sign,” referring to the tapering of the inferior oesophagus resembling the beak of a bird (Fig. 4). There are primary and secondary forms of achalasia. Primary (idiopathic) achalasia is uncommon, affecting approximately 1 in 100,000 people [11]. Although there seem to be rare familial cases, at this time the aetiology is not thought to have a genetic basis. A major cause of secondary achalasia is Chagas disease (infection with *Trypanosoma cruzi*). Secondary achalasia can also be the result of a number of conditions including eosinophilic gastroenteritis, amyloidosis, multiple endocrine neoplasia type 2 (MEN II), neurofibromatosis, sarcoidosis, and Anderson-Fabry disease.

#### Scleroderma

Scleroderma causes dilatation of the oesophagus due to impaired microvasculature leading to a pathological cascade beginning with neuronal injury. In contradistinction to achalasia, neuronal injury in scleroderma leads to denervation muscle atrophy/fibrosis, which in turn causes hypoperistalsis and loss of LES tone [12]. The decreased LES tone results in gastroesophageal reflux which in turn results in reflux oesophagitis, eventually culminating in stricture formation. This is in comparison to achalasia in which the dilatation of the oesophagus is due to increased lower oesophageal sphincter tone. The distended oesophagus and the propensity for reflux predisposes these patients to recurrent aspiration events (Fig. 3a and b). Indeed, patients with fluid within a dilated oesophagus and associated pulmonary parenchymal findings (especially bilateral dependent ground glass opacities and tree-in-bud nodules) should also be diagnosed with chronic aspiration.

#### Strictures

**Fig. 5:**
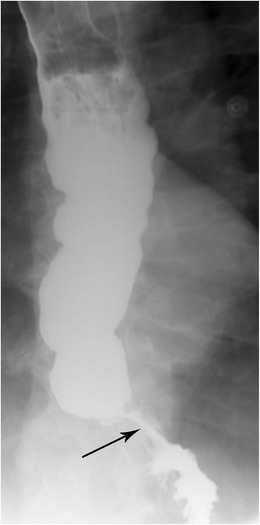
Stricture. Esophagram in left posterior oblique (LPO) projection shows an irregular short segment narrowing within the distal oesophagus consistent with a stricture. There is oesophageal dilatation proximal to the stricture

Although there is some imaging finding overlap between aetiologies, it is helpful to divide the antecedents of stricture based on the regions of the oesophagus (Fig. 5). In general, strictures in the upper and middle regions of the oesophagus tend to be caused by post-radiation changes, caustic ingestions, Barrett oesophagus, medication side effects, and various skin conditions; causes in the lower oesophagus generally include gastroesophageal reflux, scleroderma, Barrett oesophagus, and nasogastric tube placement [13]. Please note that diffuse oesophageal spasm, with its classic corkscrew appearance of the oesophagus, may mimic stricture, however these points of narrowing should not be persistent over time. Once stricture is identified, the patient should be evaluated clinically for the possibility of malignancy. In general, benign strictures are those that tend to occur in the context of long-standing dysphagia, whereas malignant strictures typically have a clear temporal course of pain and difficulty swallowing. Morphologically, malignant strictures often show irregular narrowing and constriction of the oesophageal lumen along with associated nodularity of the mucosa; in addition, the borders tend to be well defined [14]. Benign strictures, in contrast, show concentric narrowing and are more regular in appearance without associated nodularity [13].

### Luminal disorders due to foreign body impaction

**Fig. 6:**
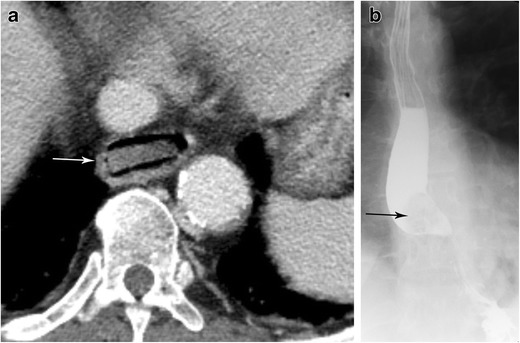
Foreign body impaction. **a** Contrast-enhanced CT of the chest in soft tissue window shows a square-shaped opacity (piece of carrot) impacted within the thoracic oesophagus just above the level of the diaphragm. **b** Esophagram in AP projection in another patient with dysphagia demonstrates a round filling defect within the midthoracic oesophagus. This was endoscopically confirmed to be an impacted hot dog

Inadvertent ingestions can lead to oesophageal obstruction with subsequent proximal dilatation (Fig. 6a and b) [15]. The physiological narrowing points of the oesophagus, as described in the anatomy and physiology section, are the most common sites of impaction. Foreign body impaction is a common occurrence that can occur in children and adults. In adults, the median age for foreign body impaction is ∼40 years of age with a significant number of cases requiring endoscopic intervention [16]. Foreign bodies that are not radiopaque may require barium swallow evaluation to identify the location of obstruction [17]. Ultimately, some foreign body impactions may be severe enough as to require surgeries. Complications from foreign body impaction are often the related to the anatomy and vascular supply of the oesophagus as well as the close proximity to other important anatomical structures in the mediastinum. In particular, it is worth mentioning that batteries, with their acidic material, may result in significant corrosive damage to the oesophagus if not promptly removed [18].

## Wall disorders

The oesophagus wall is thin (usually less than 0.3 cm) and made up of mucosa, submucosa, muscularis propria, and adventitia (but no serosa) (Fig. 2) [6, 7]. Of note, the lack of serosa is clinically relevant due to the potential of developing mediastinitis and of facilitating tumour spread. In addition, the thin wall predisposes to wall rupture and fistula formation. Normal oesophageal wall thickness varies based on the anatomic region, the degree of oesophageal dilatation, as well as the gender of the patient (males generally have a slightly thicker oesophageal wall). Pathology involving the oesophageal wall falls into several categories including wall thickening, wall thinning, wall rupture, and fistula formation.

### Wall thickening

#### Oesophagitis

**Fig. 7:**
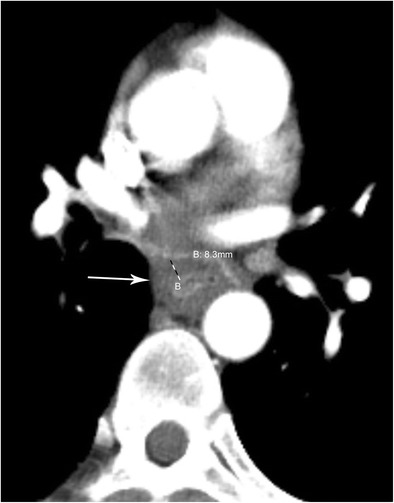
Oesophagitis. CT angiogram of the chest in soft tissue window shows circumferential oesophageal wall thickening in a patient who presented with chest pain. Please note the contrast enhancement of the mucosa which creates a hyperdense inner layer contrasting with the rest of the wall; this is an imaging feature that can be observed in gastrointestinal inflammatory diseases

Wall thickening can arise from corrosives, Crohn’s disease, reflux, infection, intubation, epidermolysis bullosa, and radiation (Fig. 7) [2]. In addition, eosinophilic oesophagitis is a less common but growing aetiology (rising in incidence along with other autoimmune diseases, asthma, and food allergies) of oesophageal thickening [19, 20]. It is thought that foreign antigens are introduced (through food or pollen) and induce eosinophilic infiltration leading to inflammation. The morbidity of the disease relates to the degree of inflammation which leads to scarring and formation of excessive fibrous tissue in the lining of the oesophagus. While it does typically respond well to steroids, some cases may require interventional dilatation or even esophagectomy.

#### Benign tumours

**Fig. 8:**
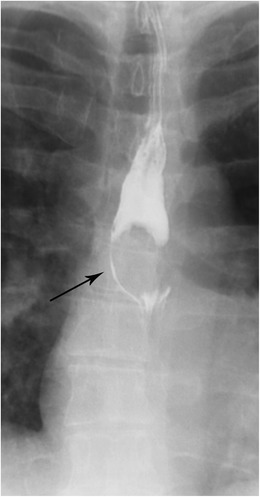
Leiomyoma. Esophagram in AP projection demonstrates a smooth, lobulated filling defect within the mid-thoracic oesophagus at the level of the carina. Notice the filling defect makes an acute angle to the oesophageal wall, suggesting that this lesion is intrinsic to the oesophagus

**Fig. 9:**
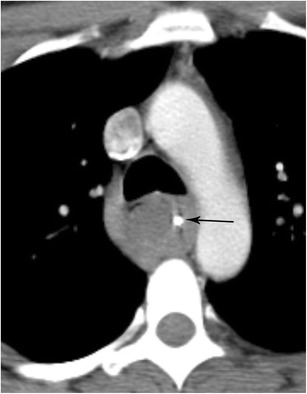
Hemangioma. CT angiogram of the chest in soft tissue window shows a round calcification within the wall of the oesophagus compatible with a phlebolith. This finding is most often seen in patients with varices, however this patient was found to have a hemangioma

Another aetiology of oesophageal wall thickening is benign tumours [21, 22]. These tumours are rare (represent less than 1% of oesophageal neoplasms) and include leiomyomas, granular cell tumours, hemangiomas, and fibroepithelial polyps. Leiomyomas are the most common benign tumour of the oesophagus. Imaging typically shows an oval-shaped intramural mass; calcifications are highly characteristic (Fig. 8) [23]. Granular cell tumours are benign tumours appearing in the gastrointestinal tract, most commonly occurring in the oesophagus (representing about a third of all such tumours). They are neural in origin and most commonly located in the distal oesophagus. Grossly, these tumours are sessile submucosal structures covered with normal mucosa [24]. Another benign tumour of the oesophagus is hemangioma. It is very rare in the oesophagus (representing only ∼3% of all benign oesophageal tumours), but when it does occur, it tends to be in the lower oesophagus. CT imaging typically shows a well-defined soft tissue mass within the oesophageal wall and phleboliths are characteristic (Fig. 9) [25].

#### Oesophageal varices

**Fig. 10:**
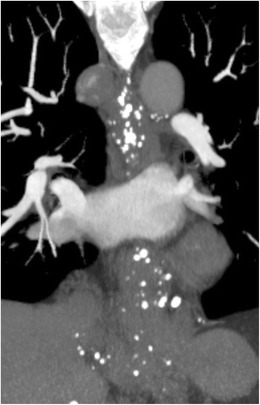
Varices. Maximum intensity projection of a CT angiogram of the chest in soft tissue window shows numerous calcifications along the oesophageal wall and portal collateral vessels in this patient with extensive uphill oesophageal varices

Oesophageal varices can be categorised as uphill (ascending from the intra-abdominal oesophagus), which are more common, or downhill (descending from the upper thoracic oesophagus) (venous anatomy reviewed in Table 1). Uphill varices are typically caused by portal hypertension, which results in collateral blood flow through the gastric and lower oesophageal veins. Uphill varices can extend superiorly to the level of the azygos vein. Downhill varices result from obstruction of the superior vena cava leading to collateral blood flow through the oesophageal veins. If the SVC obstruction is above the level of the azygos vein, the varices will extend inferiorly to the level of the carina, after which blood will drain into the azygos system. However, if the obstruction occurs below the level of the azygos vein, the varices can extend more inferiorly. Varices produce serpiginous filing defects on fluoroscopic barium studies. On CT, varices can give the appearance of a thickened oesophageal wall and phleboliths can mimic oesophageal wall calcifications, however the dilated vessels are easily identified on intravenous contrast-enhanced images (Fig. 10) [26]. Chest x-ray can show oesophageal varices as a lobular retrocardiac mass [27]. Despite their appearance, varices typically do not produce obstructive symptoms.

#### Intramural hematoma

**Fig. 11:**
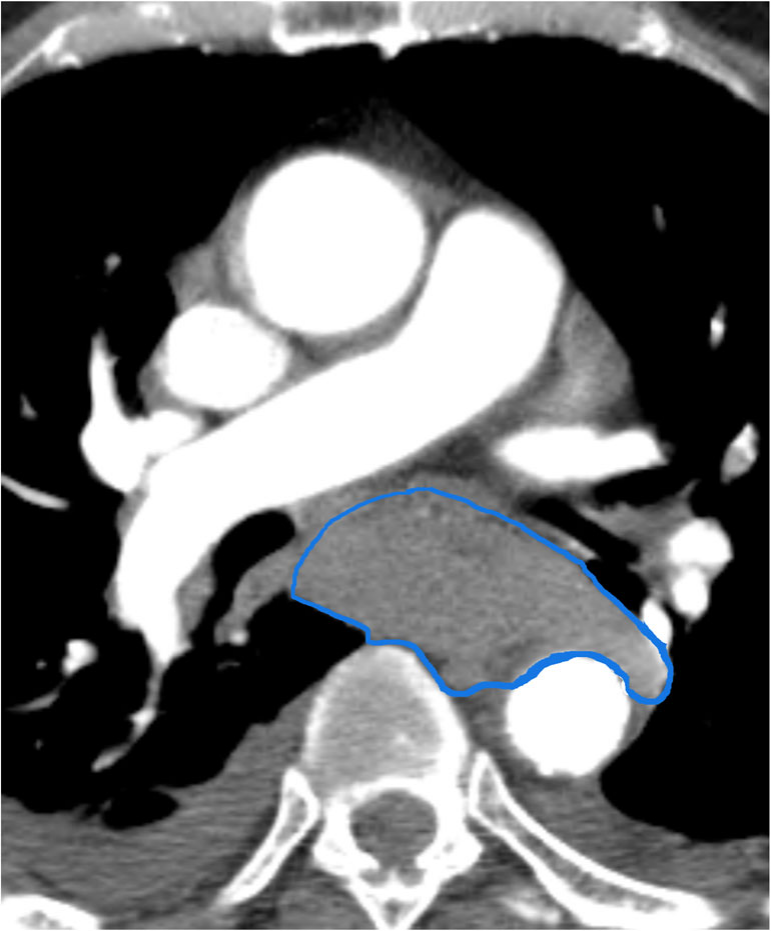
Intramural Hematoma. CT angiogram of the chest in soft tissue window shows a markedly thickened oesophageal wall that is slightly higher in density than soft tissue. The oesophageal wall partly surrounds the aorta. This patient was found to have a large intramural hematoma

In addition to spontaneous and iatrogenic hematoma formation, the many possible aetiologies of intramural hematoma include trauma, caustic oesophageal injury, and vomiting [28]. Intramural hematoma due to blunt trauma is uncommon due to the protected positioning of the oesophagus [29]. The issue in these cases is often satisfaction of search and delineating vascular, spinal cord, pulmonary (especially tracheal), pleural, and musculoskeletal abnormalities. Trauma in the oesophagus is very important since it may have implications for positioning of enteric tubes and nonspecific complaints of chest pain. Imaging will typically show an intramural fluid collection distending the walls of the oesophageal lumen which will be of intermediate density but higher than the blood pool on non-contrast imaging and will be hypodense on post-contrast imaging (Fig. 11). In addition, areas of active contrast extravasation may be present within the wall of the oesophagus or seen filling the oesophageal lumen. Patients chronically anticoagulated on warfarin are at increased risk of hematoma formation. As patients with this condition typically present with chest pain, this condition must be differentiated from acute cardiovascular disease related to the aorta, pulmonary arteries, or coronary arteries, all of which may cause bleeding in the mediastinum. Identifying the centre of the hematoma mass and knowledge of acute aortic injury can help differentiate oesophageal injury from vascular injury (Fig. 11) [30]. Intramural hematoma typically resolves in a few days or weeks without intervention, however follow-up imaging is indicated in these patients.

### Wall thinning/outpouching

The majority of oesophageal diverticula are cause by herniation of the mucosa and submucosa through the muscularis layer of the oesophagus. Thus, they are actually pseudodiverticula, since not all histological layers are involved. Diverticula are typically categorised as either pulsion or traction. Pulsion diverticula are much more common and are thought to originate due to oesophageal dysmotility. Zenker, Killian-Jamieson, and epiphrenic diverticula are all examples of pulsion diverticula. Less common are traction diverticula, which occur secondary to pulling forces on the oesophagus. They are most common in the middle oesophagus and are often secondary to inflammation (especially in the background of agents such as tuberculosis or histoplasmosis) [31]. Please note that tertiary contractions (those that are non-propulsive) may sometimes mimic traction diverticula, however should not persist over time.

#### Zenker diverticulum

**Fig. 12:**
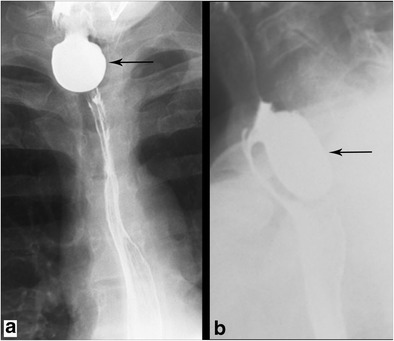
Zenker Diverticulum. Esophagram in AP (**a**) and oblique (**b**) projections demonstrates contrast filling a midline posterior outpouching of the cervical oesophageal lumen consistent with a Zenker diverticulum

This is the most common diverticulum in the oesophagus and results from herniation of the mucosa through a weak area of the cricopharyngeus muscle. Patients with these diverticula can be asymptomatic or suffer a range of symptoms including dysphagia, chronic cough, and regurgitation of food [32]. On barium esophagram, a Zenker diverticulum will present as a midline posterior oesophageal outpouching which will pool oral contrast. It is best seen in the lateral projection (Fig. 12a and b).

#### Killian-Jamieson diverticulum

**Fig. 13:**
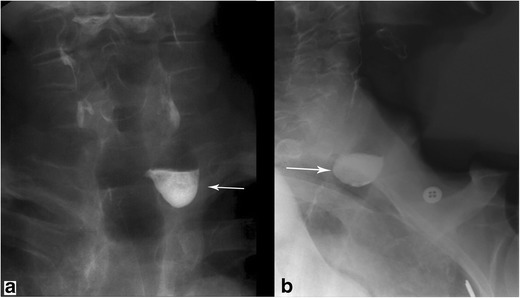
Killian-Jamieson Diverticulum. Esophagram in AP (**a**) and oblique (**b**) projections demonstrates a lateral outpouching of the lumen arising from lower cervical oesophagus consistent with a Killian-Jamieson diverticulum

In contrast to Zenker diverticula, Killian-Jamieson diverticula protrude laterally through the anterolateral wall [32, 33]. They are less common than Zenker diverticula. Barium imaging will demonstrate oral contrast pooling within a lateral outpouching off of the upper oesophagus. It is best seen on the anterior-posterior (AP) projection (Fig. 13a and b).

#### Epiphrenic diverticulum

**Fig. 14:**
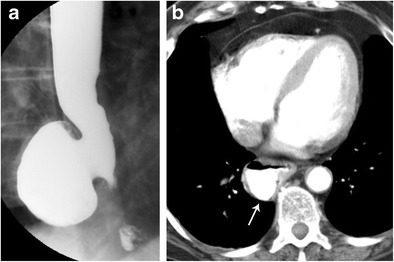
Epiphrenic Diverticulum. **a** Esophagram in oblique projection demonstrates herniation of the lumen within the lower oesophagus centred less than 10 cm from the gastroesophageal junction consistent with an epiphrenic diverticulum. **b** CT of the chest with IV and oral contrast in soft tissue window shows contrast filling an outpouching located lateral to the lower thoracic oesophagus

An epiphrenic diverticulum occurs in the distal third of the oesophagus (within 10 cm of the gastroesophageal junction) (Fig. 14a and b) [34, 35]. It is treated with diverticulectomy. Failure to treat an epiphrenic diverticulum may predispose to bleeding, aspiration, pneumonia, and/or cancer.

### Wall rupture

#### Iatrogenic perforation

**Fig. 15:**
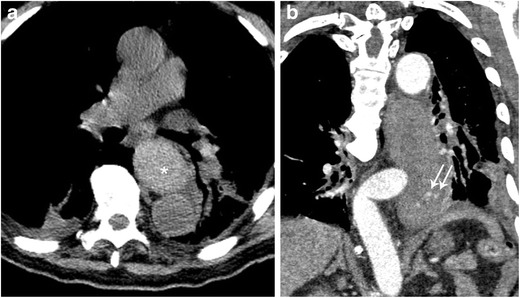
Complication of food impaction with tear of oesophagus during chicken bone extraction. **a** Pre-contrast axial image of the thorax demonstrates a markedly enlarged oesophagus which is high in density (*) when compared to the adjacent blood pool in the aorta (findings consistent with hematoma). **b** Post-contrast imaging of the same patient demonstrates areas of active contrast extravasation (arrows) within a markedly enlarged and abnormal appearing oesophagus. Clinical note, patient subsequently had multiple episodes of hematemesis

Endoscopic procedures, surgical procedures involving adjacent structures, and thermal injury during left atrial ablation can all result in iatrogenic perforation; at least half of all oesophageal perforations are estimated to be iatrogenic in nature (Fig. 15a and b) [28, 36]. Patients with perforation (whether iatrogenic or due to Boerhaave syndrome) present with sudden onset of severe epigastric pain. Typical imaging findings include pneumomediastinum, pleural effusion (left > right), and mediastinal hematoma [30].

#### Boerhaave syndrome

**Fig. 16:**
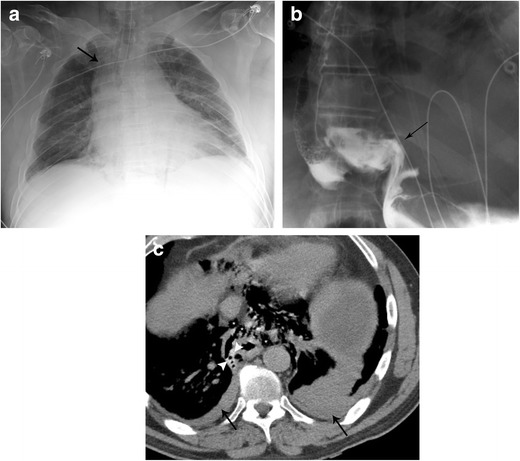
Boerhaave Syndrome. **a** AP chest radiograph shows pneumomediastinum (arrow). **b** Esophagram in AP projection demonstrates contrast extravasating from the distal oesophagus. **c** CT of the chest with oral contrast in soft tissue window demonstrates extraluminal contrast (white arrowheads), pneumomediastinum (*), and bilateral pleural effusions (arrows) in a patient with Boerhaave Syndrome

This syndrome involves a spontaneous perforation of the thoracic oesophagus (Fig. 16 a–c). Boerhaave syndrome occurs when incomplete cricopharyngeal relaxation during vomiting results in a sudden increase in intraluminal oesophageal pressure [37]. Rupture is most common in the distal left posterior wall immediately above the diaphragm (representing approximately 90% of ruptures). Treatment is immediate thoracotomy and mortality is very high without prompt intervention.

#### Mallory-weiss tears

Although it can be due to many causes, a Mallory-Weiss tear refers to a longitudinal mucosal laceration (whereas Boerhaave syndrome involves the entire wall) that typically occurs in the lower oesophagus or at the GE junction [28]. Patients commonly presenting with this condition include alcoholics and patients with eating disorders involving vomiting [38]. The pathogenesis is similar to Boerhaave syndrome. It is important to note that a mucosal laceration without transmural perforation can be radiologically occult. Typical imaging findings may include subtle extraluminal gas or haemorrhage [30].

### Fistula formation

As a general principle, the close proximity of the oesophagus to other mediastinal structures predisposes to fistula formation and secondary disease development. As the oesophagus abuts the pericardium, trachea, and aorta, fistulas to all of these structures are possible [30, 39].

#### Pericardioesophageal fistula

**Fig. 17:**
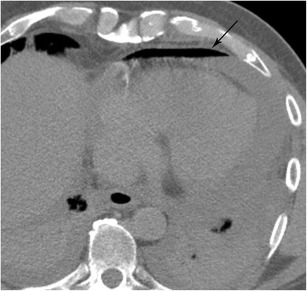
Pericardioesophageal Fistula. CT of the chest in soft tissue window demonstrates fluid and gas (arrow) within the pericardium in a patient with a confirmed pericardioesophageal fistula

**Fig. 18:**
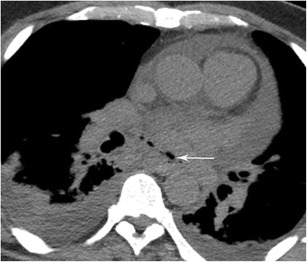
Atrioesophageal Fistula. CT of the chest in soft tissue window demonstrates thickening of the oesophageal wall with pockets of gas (arrow) within the left atrium. Additionally, there are bilateral pleural fluid collections and a pericardial effusion

Fistulas to the pericardium can occur after radiofrequency (RF) catheter ablation, although the complication rate is less than 5% (Fig. 17) [40]. Patients present with nonspecific signs 1–3 weeks after the procedure and this type of fistula is frequently lethal. Urgent intervention is necessary and may involve stents, pericardial/pleural drains, and antibiotics. RF ablation can also lead to oesophageal thermal injury and ulceration of the oesophagus. Other thoracic complications of RF ablation include oesophageal perforation, pericardial effusion/hematoma, cardiac tamponade, pulmonary vein stenosis, atrioesophageal fistula, and phrenic nerve injury (Fig. 18).

#### Tracheoesophageal fistula

**Fig. 19:**
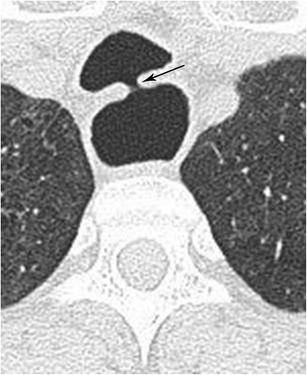
Tracheoesophageal Fistula. CT of the chest in lung window in axial plane demonstrates communication between the trachea and oesophagus (arrow) secondary to prolonged endotracheal tube intubation

Fistulas connecting the oesophagus to the trachea can be congenital or acquired. Approximately 50% of the acquired tracheoesophageal fistulas are secondary to mediastinal malignancy. Among the non-malignant causes, more than 75% are the result of endotracheal cuff-related trauma in patients subject to prolonged mechanical ventilation (Fig. 19) [41].

#### Aortoesophageal fistula

**Fig. 20:**
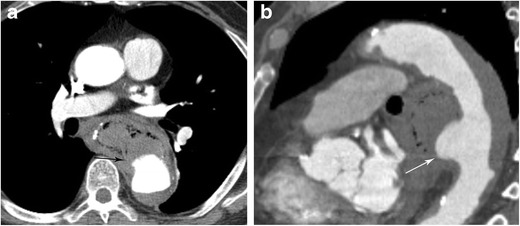
Aortoesophageal Fistula. CT angiogram of the chest in soft tissue window in axial (**a**) and sagittal (**b**) planes demonstrates extravasation of contrast from the descending thoracic aorta into the oesophagus. Soft tissue attenuation material within the oesophagus is consistent with clotted blood

Fistulas between the aorta and oesophagus are rare, although immediately life threatening (Fig. 20a and b) [42]. About two-thirds of these fistulas develop secondary to aortic aneurysm and, in general, this condition is not usually due to an underlying oesophageal abnormality [43]. Although diagnosis is not difficult on imaging, it may not be high on the differential as a cause for hemoptysis in the Emergency Department due to its rarity. Patients usually die from massive haemorrhage. Sepsis can develop from the oesophageal lesion and there can be involvement of surrounding tissues caused by infection [43]. The treatment options are case-dependent but can involve drains, open surgery, and endovascular therapy.

#### Summary

Non-malignant oesophageal disease encompasses a large variety of pathology. As discussed, benign tumours, vascular diseases, connective tissue disorders, traumatic injuries, and anatomic variants are all potentially discovered on imaging. Diagnosis of these conditions may be difficult because some of these conditions are uncommon and others may mimic cancer. However, recognizing non-malignant oesophageal diseases can be critical to appropriate and prompt medical treatment, highlighting the essential role of the radiologist in identifying this pathology on imaging.
